# *Diaporthe* species associated with the maritime grass *Festuca rubra* subsp. *pruinosa*

**DOI:** 10.3389/fmicb.2023.1105299

**Published:** 2023-02-16

**Authors:** Rufin Marie Kouipou Toghueo, Beatriz R. Vázquez de Aldana, Iñigo Zabalgogeazcoa

**Affiliations:** Plant-Microorganism Interaction Research Group, Institute of Natural Resources and Agrobiology of Salamanca, Consejo Superior de Investigaciones Científicas (IRNASA-CSIC), Salamanca, Spain

**Keywords:** grasses, *Festuca rubra subsp. pruinosa*, endophyte, multi-locus phylogeny, *Diaporthe atlantica*, *Diaporthe iberica*

## Abstract

*Festuca rubra* subsp. *pruinosa* is a perennial grass growing in sea cliffs where plants are highly exposed to salinity and marine winds, and often grow in rock fissures where soil is absent. *Diaporthe* species are one of the most abundant components of the root microbiome of this grass and several *Diaporthe* isolates have been found to produce beneficial effects in their host and other plant species of agronomic importance. In this study, 22 strains of *Diaporthe* isolated as endophytes from roots of *Festuca rubra* subsp. *pruinosa* were characterized by molecular, morphological, and biochemical analyses. Sequences of the nuclear ribosomal internal transcribed spacers (ITS), translation elongation factor 1-α (*TEF1*), beta-tubulin (*TUB*), histone-3 (*HIS*), and calmodulin (*CAL*) genes were analyzed to identify the isolates. A multi-locus phylogenetic analysis of the combined five gene regions led to the identification of two new species named *Diaporthe atlantica* and *Diaporthe iberica*. *Diaporthe atlantica* is the most abundant *Diaporthe* species in its host plant, and *Diaporthe iberica* was also isolated from *Celtica gigantea*, another grass species growing in semiarid inland habitats. An *in vitro* biochemical characterization showed that all cultures of *D. atlantica* produced indole-3-acetic acid and ammonium, and the strains of *D. iberica* produced indole 3-acetic acid, ammonium, siderophores, and cellulase. *Diaporthe atlantica is* closely related to *D. sclerotioides*, a pathogen of cucurbits, and caused a growth reduction when inoculated in cucumber, melon, and watermelon.

## 1. Introduction

The genus *Diaporthe* is highly complex, according to Index Fungorum (2022) comprises nearly 1,000 fungal names without taking into account the 984 names attributed to its asexual state *Phomopsis*. *Diaporthe* is worldwide-distributed and associated with a broad range of host plants. Some members of this genus are important pathogens, responsible for several economically significant plant diseases including stem, root and fruit rots, gummosis, cankers, leaf spots, blights, diebacks, decay, and wilts on hosts such as citrus, grapevines, soybean, peach, or sunflower, to name just a few (Udayanga et al., 2011; Marin-Felix et al., 2019). They can also colonize decaying plant tissues as saprophytes (Thongkantha et al., 2008), or live endophytically inside healthy plant tissues (Gomes et al., 2013).

As endophytes, *Diaporthe* species have been isolated from agricultural, medicinal and ornamental plants growing in a wide variety of locations and habitats (Huang et al., 2015; Dos Santos et al., 2016, 2021; Yang et al., 2018). They have also been reported as dominant taxa in fungal microbiomes of plant species like *Tectona grandis* (Murali et al., 2006), the Brazilian medicinal plants *Vochysia divergens* and *Stryphnodendron adstringens* (Noriler et al., 2018), olive trees (Martins et al., 2016), *Fagus crenata* (Tateno et al., 2015) and the grass *Festuca rubra* subsp. *pruinosa* (Pereira et al., 2019). Non-pathogenic *Diaporthe* species have been recognized as plant growth promoters (Vázquez de Aldana et al., 2021; da Silva Santos et al., 2022; Toghueo et al., 2022), and biocontrol agents (Marak et al., 2002; Dos Santos et al., 2016; Dini-Andreote, 2020; Abramczyk et al., 2022). From an industrial and pharmaceutical point of view, they are well-known producers of enzymes (Lange et al., 2021; Baluyot et al., 2022) and secondary metabolites exhibiting a wide range of activities including antimicrobial, antiviral, antioxidant, anti-inflammatory and anticancer (Chepkirui and Stadler, 2017; Xu et al., 2021). Therefore, studies aiming to analyze and better characterize *Diaporthe* species are of interest, particularly to fully comprehend not only their role as ubiquitous species in nature, but also their potential as metabolite producers, plant growth promoters, biocontrol agents, or for control measures in case of potential pathogenicity.

Previously, *Diaporthe* species were identified on the basis of morphological characteristics (e.g., colony phenotype, size, shape, and type of spores) and host specificity (Rehner and Uecker, 1994; Udayanga et al., 2011). This methodology of *Diaporthe* identification resulted in a proliferation of names in the literature. However, several studies demonstrated that morphological characters are not sufficient for species level diagnoses, and that many species of *Diaporthe* have multihost capability (Rehner and Uecker, 1994; Thompson et al., 2011, 2015; Udayanga et al., 2012). Nowadays, *Diaporthe* species are being redefined based on a polyphasic approach, employing a combination of information including morphological characteristics and multi-locus sequence data (Udayanga et al., 2012, 2014a,b; Gomes et al., 2013; Norphanphoun et al., 2022). Currently, the taxonomy of *Diaporthe* species is being resolved by means of multigene phylogenetic analyses based on sequences of the 5.8S rDNA and internal transcribed spacers (ITS1-5.8S-ITS2), translation elongation factor 1-alpha (*TEF1*), beta-tubulin (*TUB*), histone H3 (*HIS*), and calmodulin (*CAL*) genes (Udayanga et al., 2012; Gomes et al., 2013; Marin-Felix et al., 2019; Norphanphoun et al., 2022). Using this methodological approach, the delimitation of species in the genus has improved. Lately, *Diaporthe* species occurring in several host plant families have been well-characterized and sometimes reclassified, resulting in an actively evolving taxonomy with numerous novel species being described each year (Gomes et al., 2013; Dissanayake, 2017; Guarnaccia et al., 2018; Yang et al., 2018; Guo et al., 2020; Wang et al., 2021; Norphanphoun et al., 2022). Despite the attention given to pathogenic *Diaporthe* species occurring in economically important crops, more effort is needed to understand the ecology and distribution of non-pathogenic species occurring in numerous plant species.

*Festuca rubra*, commonly known as red fescue, is a perennial grass distributed across a very diverse range of habitats in the Northern Hemisphere. Red fescues are cultivated and used as turfgrasses in ornamental and sports lawns, and some cultivars have been used for phytoremediation and rehabilitation of damaged soils (Braun et al., 2020). Among its several subspecies, *Festuca rubra* subsp. *pruinosa* (*Festuca pruinosa* thereafter) is native to the Atlantic coasts of Europe and North America, where often grows in sea cliffs as a chasmophyte in rock fissures where soil is absent (Castroviejo, 2020). In this highly unhospitable habitat, plants grow with a low nutrient availability and nearly continuous exposure to salinity and desiccating winds. A previous study revealed that the roots of *Festuca pruinosa* plants have a complex endophytic fungal microbiome, and *Diaporthe* is one of its most abundant components, occurring in 54% of the plants, and at all locations analyzed (Pereira et al., 2019). The main goal of this study was to identify the endophytic *Diaporthe* species associated to roots of *Festuca pruinosa* by means of genotypic, morphological, and biochemical analyses.

## 2. Materials and methods

### 2.1. Fungal isolates

Twenty-two *Diaporthe* isolates (Table 1) obtained from surface-disinfected roots of healthy *Festuca pruinosa* plants were analyzed (Pereira et al., 2019). Plants were collected at four locations in sea cliffs in the North Atlantic coast of Spain: Torre de Hércules (43°23’09” N 8°24’23”W), Cedeira (43°40’46”N 8°01’15”W), Estaca de Bares (43°47’25”N 7°41’16”W), and San Pedro de la Rivera (43°34’43”N 6°13’17”W). In addition, we included a *Diaporthe* isolate (T6) obtained from roots of *Celtica gigantea* (= *Stipa gigantea*), a tall grass growing in dry sandy soils collected in Cuatro Calzadas, Salamanca (40°49’03”N, 5°36’47”W) (Vázquez and Devesa, 1996; Vázquez de Aldana et al., 2021).

**TABLE 1:** *Diaporthe* species, host plant, geographic origin, and GenBank accession numbers of strains used in the study. (*Festuca rubra subsp. pruinosa* = *Festuca pruinosa*).

Species	Strain	Isolation source	Origin	GenBank accession number				
				**ITS**	***CAL***	***HIS***	***TEF1***	***TUB***
*Diaporthe ambigua*	CBS 114015 (epitype)	*Pyrus communis*	South Africa	KC343010	KC343252	KC343494	KC343736	KC343978
	CBS 117167	*Aspalathus linearis*	South Africa	KC343011	KC343253	KC343495	KC343737	KC343979
*Diaporthe arecae*	CBS 161.64 (ex-isotype)	*Areca catechu*	India	KC343032	KC343274	KC343516	KC343758	KC344000
	CBS 535.75	*Citrus sp.*	Suriname	KC343033	KC343275	KC343517	KC343759	KC344001
*Diaporthe foeniculacea*	CBS 123208 (holotype of *D. neotheicola*)	*Foeniculum vulgare*	Portugal	KC343104	KC343346	KC343588	KC343830	KC344072
	CBS 123209; Di-C004/4 (ex-type of *D. neotheicola*)	*Foeniculum vulgare*	Portugal	KC343105	KC343347	KC343589	KC343831	KC344073
*Diaporthe ganjae*	CBS 180.91 = ILLS 43621 (ex-type)	*Cannabis sativa*	USA	KC343112	KC343354	KC343596	KC343838	KC344080
*Diaporthe inconspicua*	CBS 133813 (type)	*Maytenus ilicifolia*	Brazil	KC343123	KC343365	KC343607	KC343849	KC344091
	LGMF922 = CPC 20298	*Spondias mombin*	Brazil	KC343124	KC343366	KC343608	KC343850	KC344092
*Diaporthe infecunda*	CBS 133812 (type)	*Schinus terebinthifolius*	Brazil	KC343126	KC343368	KC343610	KC343852	KC344094
	LGMF918	*Schinus terebinthifolius*	Brazil	KC343132	KC343374	KC343616	KC343858	KC344100
*Diaporthe longispora*	CBS 194.36 (type of *D. strumella* var. *longispora*	*Ribes sp.*	Canada	KC343135	KC343377	KC343619	KC343861	KC344103
*Diaporthe mayteni*	CBS 133185 = CPC 20314 (ex-type)	*Maytenus ilicifolia*	Brazil	KC343139	KC343381	KC343623	KC343865	KC344107
*Diaporthe musigena*	CBS 129519 (holotype)	*Musa sp.*	Australia	KC343143	KC343385	KC343627	KC343869	KC344111
*Diaporthe pseudophoenicicola*	CBS 462.69 (ex-type)	*Phoenix dactylifera*	Spain	KC343184	KC343426	KC343668	KC343910	KC344152
	CBS 176.77	*Mangifera indica*	Iraq	KC343183	KC343425	KC343667	KC343909	KC344151
*Diaporthe sclerotioides*	CBS 296.67 (type of *P. sclerotioides*)	*Cucumis sativus*	Netherlands	KC343193	KC343435	KC343677	KC343919	KC344161
	CBS 710.76	*Cucumis sativus*	Netherlands	KC343194	KC343436	KC343678	KC343920	KC344162
*Diaporthe atlantica sp. nov*	TH2	*Festuca pruinosa*	Spain	ON159897	ON364024	ON398814	ON398836	ON364045
	TH10	*Festuca pruinosa*	Spain	ON159898	ON364025	ON398815	ON398837	ON364046
	TH21	*Festuca pruinosa*	Spain	ON159899	ON364026	ON398816	ON398838	ON364047
	TH56	*Festuca pruinosa*	Spain	ON159900	ON364027	ON398817	ON398839	ON364048
	TH71	*Festuca pruinosa*	Spain	ON159901	ON364023	ON398818	ON398840	ON364044
	TH86	*Festuca pruinosa*	Spain	ON159903	ON364029	ON398820	ON398842	ON364050
	TH91	*Festuca pruinosa*	Spain	ON159904	ON364030	ON398821	ON398843	ON364051
	TH151	*Festuca pruinosa*	Spain	ON159905	ON364031	ON398822	ON398844	ON364052
	TH158	*Festuca pruinosa*	Spain	ON159906	ON364032	ON398823	ON398845	ON364053
	EB4	*Festuca pruinosa*	Spain	OM944045	ON018830	ON018832	ON018831	ON018829
	EB11	*Festuca pruinosa*	Spain	ON159889	ON364015	ON398806	ON398827	ON364036
	EB12	*Festuca pruinosa*	Spain	ON159890	ON364016	ON398807	ON398828	ON364037
	CD39	*Festuca pruinosa*	Spain	ON159887	ON364013	ON398804	ON398825	ON364034
	CD87	*Festuca pruinosa*	Spain	ON159888	ON364014	ON398805	ON398826	ON364035
	SP11 = CECT 21217 (holotype)	*Festuca pruinosa*	Spain	ON159893	ON364019	ON398810	ON398831	ON364040
	SP45	*Festuca pruinosa*	Spain	ON159894	ON364020	ON398811	ON398832	ON364041
	SP130	*Festuca pruinosa*	Spain	ON159895	ON364021	ON398812	ON398833	ON364042
	SP131	*Festuca pruinosa*	Spain	ON159896	ON364022	ON398813	ON398834	ON364043
*Diaporthe iberica sp. nov*	S32	*Festuca pruinosa*	Spain	ON159892	ON364018	ON398809	ON398830	ON364039
	T6	*Celtica gigantea*	Spain	MT645115	ON364033	ON398824	ON398835	ON364054
	TH77 = CECT 21218 (holotype)	*Festuca pruinosa*	Spain	ON159902	ON364028	ON398819	ON398841	ON364049
*Diaporthe* sp.1	EB73 = CECT 21219	*Festuca pruinosa*	Spain	ON159891	ON364017	ON398808	ON398829	ON364038
*Diaporthella corylina*	CBS 121124 = AR 4131	*Corylus sp.*	China	KC343004	KC343246	KC343488	KC343730	KC343972

Type cultures are indicated in bold. CBS, CBS FUNGAL Biodiversity Centre, Utrecht, The Netherlands; CECT, Spanish type culture collection; CPC, Collection Pedro Crous, housed at CBS; ILLS, Illinois Natural History Survey Fungarium; LGMF, Culture collection of Laboratory of Genetics of Microorganisms, Federal University of Parana, Curitiba, Brazil; *TUB*, partial beta-tubulin gene; *CAL*, partial calmodulin gene; *HIS*, partial histone H3 gene; ITS, ITS1-5.8S rDNA-ITS2 internal transcribed spacer region; *TEF1*, partial translation elongation factor 1-alpha gene. Isolates marked with bold are ex-type or ex-epitype strains.

### 2.2. DNA isolation, PCR amplification, and sequencing

Fungal DNA was extracted and amplified from a small amount of mycelium scraped from seven-day-old potato dextrose agar (PDA) cultures using Extract-N-Amp Plant Tissue PCR kits (Merck). The oligonucleotide primers and PCR protocols used for the amplification of the different fungal genes are listed in Supplementary Table 1. After amplification, PCR amplicons were sequenced in both directions at the DNA sequencing service of the University of Salamanca (Spain). New sequences generated in this study were deposited at the GenBank nucleotide database (Table 1).

### 2.3. Phylogenetic analysis

To establish the identity of fungal isolates at species level, phylogenetic analyses based on sequences of five genes (ITS, *TUB*, *CAL*, *TEF1*, and *HIS*) were conducted. To determine which *Diaporthe* taxa were closest to our fungal isolates, a concatenated sequence of the five loci was aligned with a similar set of 243 sequences belonging to 95 *Diaporthe* species (TreeBASE, study S13943; Gomes et al., 2013). The alignment was performed using MAFFT (Katoh and Standley, 2013) with default settings, and manually adjusted with MEGA v. 7 (Kumar et al., 2016). Then, a Maximum Likelihood (ML) phylogenetic tree was made using MEGA v.7 to identify the clades to which our isolates belonged. Afterwards, more restricted phylogenetic trees were made using the sequences of the species included in these major clades. These phylogenetic analyses were based on ML for all the individual loci, and on both ML and Bayesian Inference (BI) for the concatenated sequence of the five loci. The best-fit models for each gene and the concatenated set were determined using MEGA v. 7 and incorporated into the analyses. For the BI, MrBayes v. 3.2.7 (Ronquist et al., 2012) was used to generate the phylogenetic trees under optimal criteria per data partition. The Markov Chain Monte Carlo analysis of four chains ran for 20 000 000 generations and started in parallel from a random tree topology and lasted until the average standard deviation of split frequencies was below 0.01. Trees were saved each 10 000 generations. The first 25% of saved trees were discarded as the burn-in phase and the posterior probabilities were determined from the remaining trees. The resulting phylogenetic tree was printed with Geneious v. 5.5.4 (Drummond et al., 2011). A ML tree for the concatenated sequence of the five loci was based on the Hasegawa-Kishino-Yano model with gamma correction. For other parameters, default settings were used. ML analyses were performed in MEGA v.7 with the tree bisection and reconnection algorithm, where gaps were treated as missing data. The robustness of the topology was evaluated by 1,000 bootstrap replications. *Diaporthella corylina* (CBS 121124) was used as an outgroup. Holotypes were preserved as metabolically inactive cultures at the Spanish Type Culture Collection (CECT). Nomenclatural novelties and descriptions were deposited at the CECT and MycoBank (MB).

### 2.4. Genetic diversity analysis

The 18 cultures of *D. atlantica* available were used for an analysis of its genetic diversity. For the samples investigated, diversity indices were calculated for each gene and the combined sequence dataset. Parameters such as the Tajima’s D (Tajima, 1989), the minimum numbers of recombination events (Rm) (Hudson and Kaplan, 1985), the total number of haplotypes (H), haplotype diversity (Hd), Watterson’s θ (θw), the number of segregating sites (S), and the average nucleotide diversity (pi) were calculated using the DnaSP v. 6.12 software (Librado and Rozas, 2009). To overcome the population size effects, Hd, θw, and pi were calculated after 1,000 repetitions, and the median estimate was recorded for each parameter.

### 2.5. Morphological characters

Agar plugs (6 mm diameter) from the edge of actively growing cultures on PDA were transferred to 9 cm diameter Petri dishes containing one of the following culture media: malt extract agar (MEA), PDA, water agar supplemented with sterile pine needles (PNA) (Smith et al., 1996) or with sterile pieces of *Festuca pruinosa* leaves (FLA). Plates were incubated at 21–22°C under a 12 h/12 h near-ultraviolet light/darkness cycle to induce sporulation as described by Gomes et al. (2013). Cultures were examined periodically for the development of ascomata or conidiomata. Colony diameters were determined on PDA cultures grown at 22–25°C in darkness after 3 days, while colony colors were described after 14 days using the charts of Rayner (1970). For microscopy, fungal structures were mounted in distilled water, and measurements determined for 30 conidia and other structures.

### 2.6. Biochemical properties of *Diaporthe* strains

The 22 *Diaporthe* strains were investigated for their metabolic activity *in vitro* as follows. Ammonium production was determined both qualitatively and quantitatively, as described by Cappuccino and Sherman (1998). Siderophore activity was determined using the chrome azurol S agar plate assay as described by Schwyn and Neilands (1987). The production of indole-3-acetic acid (IAA) was determined quantitatively using both spectrophotometric (Gordon and Weber, 1951) and chromatographic (Patel et al., 2018) assays. The ability of *Diaporthe* strains to produce amylase, cellulase and protease was analyzed *in vitro* in the supplemented media described by Hankin and Anagnostakis (1975). The phosphate solubilizing ability was determined using Pikovskaya’s agar medium, as described by Katznelson and Bose (1959).

### 2.7. Pathogenicity assays

Because of the close relatedness of the new *Diaporthe* species to *D. sclerotioides*, a well-known pathogen of cucurbits (Shishido et al., 2006, 2014), the pathogenicity of *Diaporthe* strains EB4 (*D. atlantica*) and S32 (*D. iberica*) was tested on cucumber (*Cucumis sativus*) cv. Ashley, melon (*Cucumis melo*) cv. Piñonet, and watermelon (*Citrullus lanatus*) cv. Crimson Sweet.

Mycelial inoculum of both *Diaporthe* strains was produced in 3-week old sugar beet pulp cultures (Vázquez de Aldana et al., 2020), and seedlings of the three cucurbits were obtained after seed germination in sterile vermiculite. Fourteen day old seedlings were transplanted to 200 mL pots containing a substrate consisting of seven volumes of a mixture of peat and perlite (1:1; v:v) previously treated at 80°C for 12 h in a forced air oven, and one volume of *Diaporthe* inoculum. Control seedlings were transplanted to substrate containing only the peat and perlite mixture. One seedling was transplanted to each pot and each treatment was replicated in six pots. The plants were maintained in a greenhouse, being tap watered on demand for a period of 40 days. After this time, the plants were harvested, the roots were cleaned with tap water and inspected for disease symptoms such as dark lesions. Dry weights of roots and aboveground tissues were measured, and differences among treatments were tested by means of an analysis of variance (ANOVA). Differences among means were tested with the Holm-Sidak method. Statistical calculations were made with SigmaPlot software v. 14.5.

## 3. Results

### 3.1. Phylogenetic analyses

A preliminary analysis of the concatenated sequences of the 22 *Diaporthe* strains from *Festuca pruinosa* aligned with 243 sequences belonging to 95 *Diaporthe* species (Gomes et al., 2013) allowed to identify the clades to which the Festuca isolates belonged (Supplementary Figure 1). Thereafter, we reviewed other multilocus taxonomic studies (Dissanayake et al., 2017; Gao et al., 2017; Guarnaccia et al., 2018; Guo et al., 2020; Norphanphoun et al., 2022), to search for other *Diaporthe* species closely related to the identified clades that were not included in Gomes et al. (2013). As a result, *Diaporthe columnaris* and *D. cyatheae* (Norphanphoun et al., 2022), were found to belong to the same clade as our endophytic isolates. Due to the lack of all five loci for these two species, they were not included in the multilocus tree. However, available loci were included in the single locus phylogeny to support the classification of our fungal isolates (Supplementary Figures 2–5).

**FIGURE 1:**
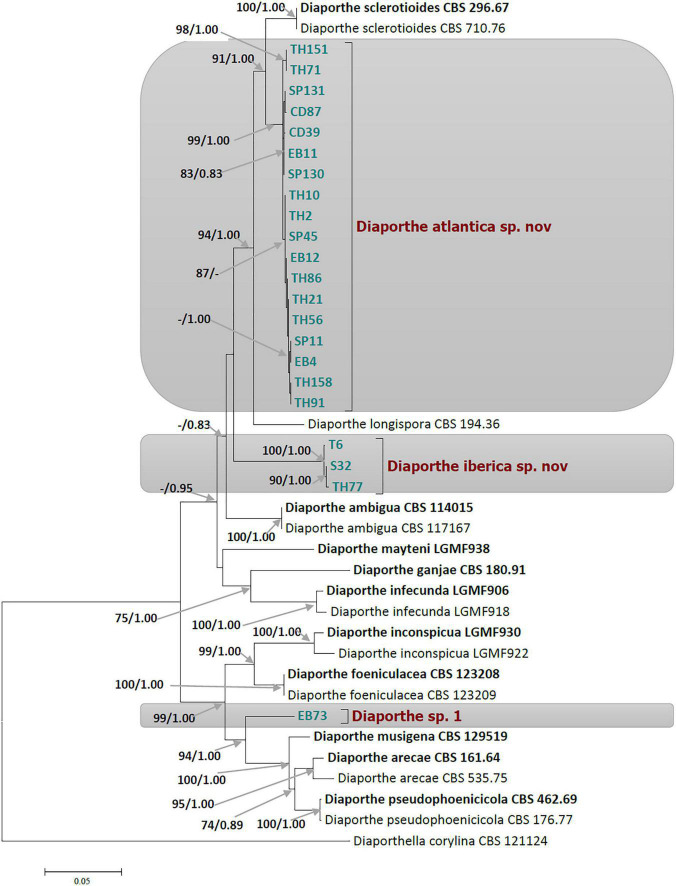
Phylogram of *Diaporthe* resulting from a maximum likelihood analysis based on a combined matrix of ITS, *TUB*, *CAL*, *TEF1*, and *HIS3*. Numbers above the branches indicate ML bootstraps (left, ML BS ≥ 70%) and Bayesian Posterior Probabilities (right, PP ≥ 0.8). The tree is rooted with *Diaporthella corylina.* Isolates from present study are marked in colors. The scale bar represents the expected changes per site. The ex-type strains are Bold and strains from this study in green.

Ultimately, a set of 11 *Diaporthe* species closely related to the endophytic *Festuca pruinosa* isolates plus *Diaporthella corylina* (outgroup) were selected for a more restricted multilocus phylogenetic analysis (Table 1). A total of 2,786 characters including gaps were included in this phylogenetic analysis, 1,368 of these were conserved and 981 were variable, 649 of which were parsimony informative. The Bayesian analysis of the combined sequences of the five loci was based on the Hasegawa-Kishino-Yano substitution model with gamma distributed rate variation among sites (Table 2) and all partitions had Dirichlet base frequencies. The Bayesian analysis lasted 20 000 000 generations resulting in an average standard deviation of split frequencies of 0.001683. The consensus tree was generated and the posterior probabilities were calculated from the 27 443 trees left after discarding the first 25% for burn-in. The topologies resulting from ML and BI analyses of the concatenated dataset were congruent. Bayesian posterior probability (PP ≥ 0.8) and Maximum likelihood bootstrap values (ML ≥ 70) were obtained for the dendrogram nodes (Figure 1).

**TABLE 2:** Nucleotide substitution models used in the phylogenetic analyses.

Loci/Genes	Analysis	Best-fit model
ITS	ML	Kimura 2-parameter
TUB	ML	Hasegawa-Kishino-Yano
TEF1	ML	Kimura 2-parameter
HIS	ML	Tamura-Nei
CAL	ML	Tamura 3-parameter
Concatenated loci	ML/BI	Hasegawa-Kishino-Yano

BI, Bayesian inference; ML, maximum likelihood.

The 22 *Diaporthe* isolates from *Festuca pruinosa* were set apart from known species, and clustered together to create two novel clades statistically well-supported by ML and PP values. In the multilocus tree, 18 *Festuca pruinosa* isolates clustered together within a well-supported clade (ML/PP = 99/1.00) significantly distinct from *D. sclerotioides*, its closest known species (Figure 1). This new species was named *Diaporthe atlantica* sp. nov. Moreover, the single locus phylogeny of the five genes shows that *D. atlantica* was closer but separated from *D. sclerotioides* (Supplementary Figures 2–7) and also differed from *D. columnaris* in the ITS and *TEF1* trees (Supplementary Figures 2, 4).

Three isolates (S32, TH77, and T6) clustered together to form a new clade (ML/PP = 100/1.00) unrelated to other known *Diaporthe* species, and was named *Diaporthe iberica* sp. nov. Two of these strains were isolated from maritime populations of *Festuca pruinosa*, but strain T6 was isolated several hundred km inland from roots of *Celtica gigantea*. Interestingly, *Festuca pruinosa* strains S32 and TH77 were genotypically closer than strain T6. In addition, the single locus analysis of each of the five genes consistently differentiated the clade formed by *D. iberica* strains (S32, TH77, and T6) from all known species included in the analysis (Supplementary Figures 2–6).

The single and multilocus phylogenetic analyses also showed that the strain EB73 was well-separated from closely related species (*D. musigena*, *D. arecae*, and *D. foeniculacae*), and might as well belong to a yet undescribed *Diaporthe* species. As we currently have only a single strain of this taxon, further investigations will have to await further collections.

### 3.2. Taxonomy

Based on the multi-locus phylogeny and their morphology, the 22 strains from *Festuca pruinosa* were assigned to two newly described taxa, plus a yet undescribed species. All species studied in culture are characterized below.

**FIGURE 2:**
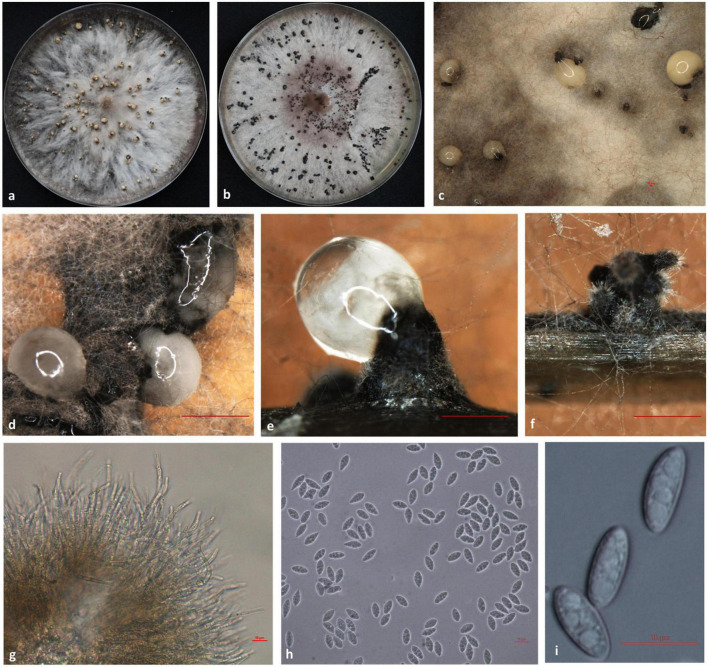
Morphological characteristics of *Diaporthe atlantica*. Colony appearance on potato dextrose agar (PDA) **(a)** and malt extract agar (MEA) **(b)**; conidiomata with conidial droplets on PDA **(c,d)**; conidiomata on pine needle **(e)** and *Festuca pruinosa* leaves **(f)**; conidiogenous cells **(g)**; α-conidia **(h,i)**. Scale bar: panels **(d–f)** = 100 μm; panels **(c,g–i)** = 10 μm.

*Diaporthe atlantica* Toghueo, Vazq-Alda and Zabalgo, sp. nov.; Figure 2 and Supplementary Figure 8.

MycoBank number: MB845433.

Etymology: Named after the Atlantic ocean, in whose coastal cliffs is found *Festuca rubra* subsp. *pruinosa*, the plant host of this species.

Description: Pycnidial conidiomata globose or irregular, black scattered or aggregated, and irregularly distributed over agar surfaces, with white or cream conidial droplets (50–160 μm) exuding from the ostioles. Conidiophores hyaline, terminal and lateral, cylindrical, 1-3-septate, densely aggregated, 14.3–30.9 × 2.5–4.1 μm, (mean ± SD = 21.84 ± 6.07×3.11 ± 0.61 μm). Conidiogenous cells [4.8–15.1 × 1.9–6.6 μm, (mean ± SD = 9.36 ± 3.84 × 4.44 ± 1.9μm)] hyaline, subcylindrical and filiform, straight to curved, terminal and lateral, with slight taper toward the apex. Alpha conidia were fusiform, hyaline, ellipsoidal, aseptate, rounded at each end, 8.7–12.3 × 3.3–5 μm, (mean ± SD = 10.29 ± 0.9 × 4.3 ± 0.42 μm). Beta and gamma conidia were not observed.

Culture characteristics: Colonies on PDA were white at first, with flattened mycelium and becoming dirty white and umber over time with dark black pycnidia bearing cream conidial droplets distributed over the agar surface. The colony margins had petaloidlike shapes. The reverse was graphite gray with black spots uniformly distributed. On MEA, colonies were white at first with flattened mycelium and becoming brownish over time. Dark black and sterile stromata abundantly distributed over the mycelium surface were observed. The colony diameter was 24–30 mm after 3 days at 25°C.

Specimens examined: SPAIN, Asturias, San Pedro de la Rivera (43°34’43”N, 6°13’17”W), from roots of *Festuca rubra* subsp. *pruinosa*, Mar. 2016, E. Pereira, holotype SP11 (CECT 21217); A Coruña, Cedeira, from roots of *Festuca pruinosa*, Mar. 2016, E. Pereira, strains CD39, CD87; A Coruña, Estaca de Bares, from roots of *Festuca pruinosa*, Mar. 2016, E. Pereira strains EB4, EB11, and EB12; Asturias, San Pedro de la Rivera, from roots of *Festuca pruinosa*, Mar. 2016, E. Pereira, strains SP45, SP130, and SP131; A Coruña, Torre de Hercules, from roots of *Festuca pruinosa*, Mar. 2016, E. Pereira, strains TH2, TH10, TH21, TH56, TH71, TH86, TH91, TH151, and TH158.

Host/Habitat: *Festuca rubra* subsp. *pruinosa* grows on rocky sea cliffs in the Atlantic coasts of Europe. In this habitat, soil and nutrients are very limited, and exposure to salinity is continuous.

Notes: *Diaporthe atlantica* was isolated as an endophyte from surface-disinfected roots of *Festuca pruinosa*. This species was described from a set of 18 strains among which only four produced spores, and this occurred on PNA medium. This species was found in plants from all the four locations investigated. *In vitro*, all 18 strains investigated produced indole-3-acetic acid (IAA), and ammonium, while 16 produced siderophores and cellulase, 13 solubilized phosphate and 11 produced amylase. *Diaporthe atlantica* is phylogenetically close but clearly differentiated from *D. sclerotioides* and *D. columnaris* two species known as pathogens of cucurbits (Shishido et al., 2014) and lingonberry (Farr et al., 2002), respectively. Morphologically *D. atlantica* cannot be reliably separated from *D. sclerotioides* due to overlaps in characteristics such as the culture phenotype or spore shape and size. However, *D. atlantica* is morphologically distant from *D. columnaris* by the size of alpha conidia, and the shape and size of conidiogenous cells (Farr et al., 2002). On the other hand, *D. atlantica* differs from *D. sclerotioides* by 110 nucleotides in the concatenated sequence alignment, 45 of which were distinct in the *TUB* region, 44 in the *TEF1* region, 10 in the ITS region, 9 in the *HIS* region, and 2 in the *CAL* region and differs from *D. columnaris* by 2 and 13 nucleotides in the ITS and *TEF1* regions respectively. Furthermore, a phylogenetic analysis based on the ITS sequences of all *D. atlantica* strains and those of thirteen isolates of *D. sclerotioides* retrieved from GenBank revealed a clear separation between both species. In the ML tree obtained, all *D. atlantica* strains clustered together in a well-supported clade (ML = 70; Supplementary Figure 7) clearly separated from the *D. sclerotioides* isolates (ML = 99; Supplementary Figure 7). Inoculation of *D. atlantica* strain EB4 in cucumber, melon, and watermelon plants did not cause root necrosis or seedling wilt, as *D. sclerotioides* does (Shishido et al., 2014), but shoot and root growth was delayed in the three plant species.

**FIGURE 3:**
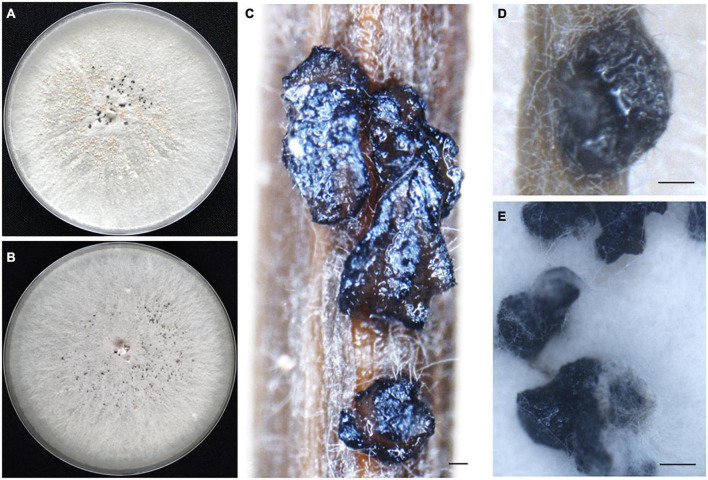
Morphological characteristics of *Diaporthe iberica*. Front view of colony appearance on potato dextrose agar (PDA) **(A)** and malt extract agar (MEA) **(B)**; sterile stromata on pine needle **(C)** and *F. pruinosa* leaf **(D)** and on the surface of PDA **(E)**; Scale bar: panels **(C–E)** = 100 μm.

*Diaporthe iberica* Toghueo, Vazq-Alda and Zabalgo, sp. nov.; Figure 3 and Supplementary Figure 8.

MycoBank number: MB845435.

Etymology: Named after the Iberian Peninsula, where isolates were obtained in different habitats and host plant species.

Description: Sterile stromata were globose or irregular, dark black, irregularly distributed over the agar surface, scattered or aggregated and exposed on the surface of *Festuca rubra* leaves and pine needles. Alpha, beta and gamma conidia were not observed.

Culture characteristics: Colonies on PDA form flattened and white mycelium. Dark sterile stromata uniformly distributed over the agar plate emerged after 2 weeks at 25°C. On MEA, colonies with flattened and white gray mycelium with dark stromata were uniformly distributed over the agar plate. The colony diameter was 22–31 mm after 3 days at 25°C. The dark stromata remained sterile on PDA, MEA PNA, and FLA after 4 months in culture at 25°C, and after 2 weeks of continuous exposure to UV light.

Specimens examined: SPAIN, A Coruña, Torre de Hercules (43°23’09”N, 8°24’23”W), from roots of *Festuca rubra* subsp. *pruinosa*, Mar. 2016, E. Pereira, holotype TH77 (CECT 21218); Asturias, San Pedro de la Rivera, from roots of *Festuca pruinosa*, Mar. 2016, E. Pereira, strain S32; Salamanca, Cuatro Calzadas, from roots of *Celtica gigantea*, Mar. 2015, I. Zabalgogeazcoa, strain T6.

Host/Habitat: *Festuca pruinosa* grows on rocky sea cliffs, with poor nutrient availability, and continuous exposure to salinity, while *Celtica gigantea* grows in nutrient-poor sandy soils in semiarid habitats in the southwestern Iberian peninsula (Vázquez and Devesa, 1996).

Notes: The three isolates of *Diaporthe iberica* studied form a clade with high support (ML/PP = 100/1.00), distinct from other known *Diaporthe* species. This new taxon can colonize at least two distinct host grasses, *Festuca pruinosa* and *Celtica gigantea*, growing in very different habitats. *In vitro*, all three strains produced IAA, ammonium, siderophores and cellulase.

*Diaporthe* sp. 1

Specimen examined: SPAIN, A Coruña, Estaca de Bares (43°47’25”N, 7°41’16”W), from roots of *Festuca pruinosa*, Mar. 2016, E. Pereira, strain EB73 (CECT 21219).

**FIGURE 4:**
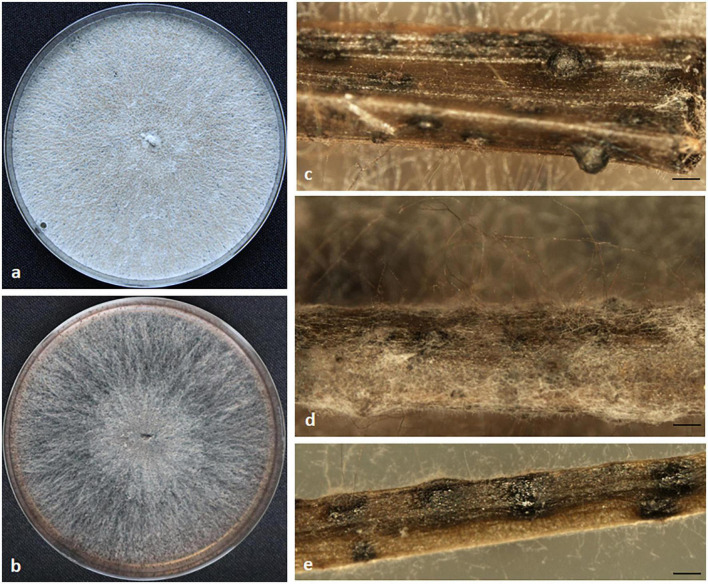
Morphological characteristics of *Diaporthe* sp. 1. Colony appearance on potato dextrose agar (PDA) **(a)** and malt extract agar (MEA) **(b)**; sterile conidiomata on pine needle **(c,d)** and *Festuca pruinosa* leaves **(e)**. Scale bar: panels **(c–e)** = 100 μm.

Culture characteristics: Colonies on PDA form white gray mycelium at first and become dark over time. On MEA, light brown aerial mycelium, cottony and less abundant. The colony diameter was 26 mm after 3 day at 25°C (Figure 4).

Host/Habitat: *Festuca rubra* subsp. *pruinosa* grows on rocky sea cliffs in the Atlantic coasts of Europe. Soil and nutrients are very limited, and exposure to salinity is continuous in this habitat.

Notes: Based on phylogenetic data, this endophytic isolate might belong to an undescribed species. Cultures were sterile on various media including PDA, MEA, PNA, and FLA at varied conditions. *In vitro*, strain EB73 produced IAA, ammonium, siderophores, cellulase and solubilized phosphate.

### 3.3. Genetic diversity of *Diaporthe atlantica*

The genetic diversity of *Diaporthe atlantica* was estimated on the basis of its 18 individuals (Table 3). The calculated haplotype diversity of *D. atlantica* was higher than 0.5 for TEF1, CAL, HIS, TUB and the combined data set, reflecting a high genetic diversity of the population analyzed (Nei and Tajima, 1981; Stumpf, 2004).

**TABLE 3:** Polymorphism and genetic diversity parameters of *Diaporthe atlantica* strains associated with *Festuca rubra* subsp. *pruinosa*.

Gene	*n*	bp	θ _w_	*S*	*H*	Hd	pi	*k*	TD	Rm
ITS	18	423	0.581	2	2	0.209	0.00099	0.418	−0.68482	0.0
*TUB*	18	709	2.035	7	7	0.869	0.00256	1.817	−0.36375	0.0
*HIS*	18	386	2.907	10	9	0.895	0.00598	2.307	−0.74337	0.0
*TEF1*	18	230	4.070	14	11	0.922	0.01998	4.595	0.20324	2.0
*CAL*	18	312	0.581	2	3	0.569	0.00297	0.928	1.45481	0.0
Combined	18	2061	8.722	30	17	0.987	0.00451	9.294	0.12562	4.0

n, sample size; bp, total number of sites; S, number of segregating/polymorphic sites (parsimony informative sites); H, number of alleles; Hd, haplotype (allelic) diversity; pi, average nucleotide diversity; k, average number of nucleotide differences; TD, Tajima’s D (TD); θ_w_, Watterson’s theta; Rm, minimum number of recombination events. TD was not significant (*P* > 0.10) for all the genes analyzed.

Overall, 17 haplotypes were detected in the concatenated gene sequence, while eleven, nine, seven, three, and two haplotypes were detected in TEF1, HIS, TUB, CAL, and ITS sequences respectively (Table 4). The only common haplotype, H-9, was shared by two strains obtained from different localities, Estaca de Bares (A Coruña) and San Pedro de la Rivera (Asturias). The haplotypes ITS-H1 (*n* = 16), CAL-H2 (*n* = 11), HIS-H6 (*n* = 5), TEF1-H4 (*n* = 4) and TUB-H5 (*n* = 5) were the most frequent for each gene. The highest degree of sequence polymorphism (pi = 0.01998) was observed for TEF1, with 11 haplotypes. Details of polymorphism at each locus and unique sequences types for each strain are available in Supplementary Table 2. In general, this analysis suggests that the populations of *Diaporthe atlantica* are genotypically diverse, but clonality exists in them.

**TABLE 4:** Haplotypes of *Diaporthe atlantica* strains isolated from roots of *Festuca rubra* subsp. *pruinosa*.

Strain	Location	Single locus haplotype					Five-locus haplotype
		**ITS**	***TUB***	***HIS***	***TEF1*-α**	***CAL***	
CD39	CED	ITS-H1	*tub*-H1	*his*-H7	*tef*1-H1	*Cal*-H1	Haplotype 1
TH2	TDH	ITS-H1	*tub*-H5	*his*-H9	*tef*1-H7	*Cal*-H2	Haplotype 2
TH10	TDH	ITS-H1	*tub*-H5	*his*-H8	*tef*1-H8	*Cal*-H2	Haplotype 3
TH21	TDH	ITS-H1	*tub*-H5	*his*-H6	*tef*1-H8	*Cal*-H2	Haplotype 4
TH86	TDH	ITS-H1	*tub*-H3	*his*-H6	*tef*1-H8	*Cal*-H2	Haplotype 5
TH91	TDH	ITS-H1	*tub*-H7	*his*-H4	*tef*1-H11	*Cal*-H2	Haplotype 6
SP45	SPR	ITS-H1	*tub*-H5	*his*-H3	*tef*1-H6	*Cal*-H2	Haplotype 7
CD87	CED	ITS-H1	*tub*-H2	*his*-H5	*tef*1-H2	*Cal*-H1	Haplotype 8
EB4	EDB	ITS-H1	*tub*-H4	*his*-H6	*tef*1-H3	*Cal*-H2	Haplotype 9
SP11	SPR	ITS-H1	*tub*-H4	*his*-H6	*tef*1-H3	*Cal*-H2	Haplotype 9
EB11	EDB	ITS-H1	*tub*-H1	*his*-H1	*tef*1-H4	*Cal*-H1	Haplotype 10
SP130	SPR	ITS-H1	*tub*-H1	*his*-H7	*tef*1-H4	*Cal*-H1	Haplotype 11
SP131	SPR	ITS-H1	*tub*-H1	*his*-H5	*tef*1-H3	*Cal*-H1	Haplotype 12
TH71	TDH	ITS-H2	*tub*-H6	*his*-H1	*tef*1-H10	*Cal*-H3	Haplotype 13
TH151	TDH	ITS-H2	*tub*-H6	*his*-H1	*tef*1-H4	*Cal*-H3	Haplotype 14
TH56	TDH	ITS-H1	*tub*-H5	*his*-H6	*tef*1-H9	*Cal*-H2	Haplotype 15
TH158	TDH	ITS-H1	*tub*-H7	*his*-H4	*tef*1-H4	*Cal*-H2	Haplotype 16
EB12	EDB	ITS-H1	*tub*-H3	*his*-H2	*tef*1-H5	*Cal*-H2	Haplotype 17

The haplotypes observed at each sequenced locus for each strain analyzed (*n* = 18), and the resulting multilocus haplotype are shown. CED, Cedeira; EDB, Estaca de Bares; SPR, San Pedro de la Rivera; TDH, Torre de Hércules; *CAL*, partial sequence of the gene coding for calmodulin; *HIS*, Histone H3; ITS, ITS1-5.8S rDNA-ITS2 internal transcribed spacer region; *TEF1*, partial sequence of the gene coding for translation elongation factor 1-α; *TUB*, partial sequence of the gene coding for β-tubulin.

### 3.4. Biochemical characteristics of endophytic *Diaporthe* species

**FIGURE 5:**
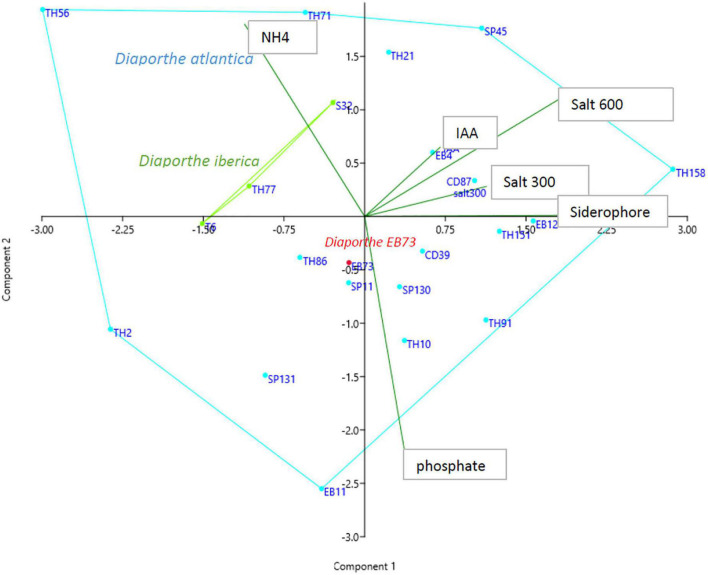
Principal component analysis of *in vitro* characteristic of *Diaporthe* strains isolated from *Festuca pruinosa*.

All 22 *Diaporthe* strains produced ammonium in peptone water and IAA in potato dextrose broth. On agar-supplemented media, 20 strains were able to produce siderophores and cellulase, 15 solubilized phosphate, 13 amylase, and 5 protease (Supplementary Table 3). A principal component analysis (PCA) was performed on the whole dataset to investigate if there is a relationship between *Diaporthe* species and some biochemical characteristics. The PCA showed that components I and II accounted for 62% of the total variance. The three *D. iberica* strains (T6, TH77, and S32) clustered in close proximity, within the sparse distribution of *D. atlantica* strains (Figure 5).

### 3.5. Pathogenicity of *Diaporthe*

**FIGURE 6:**
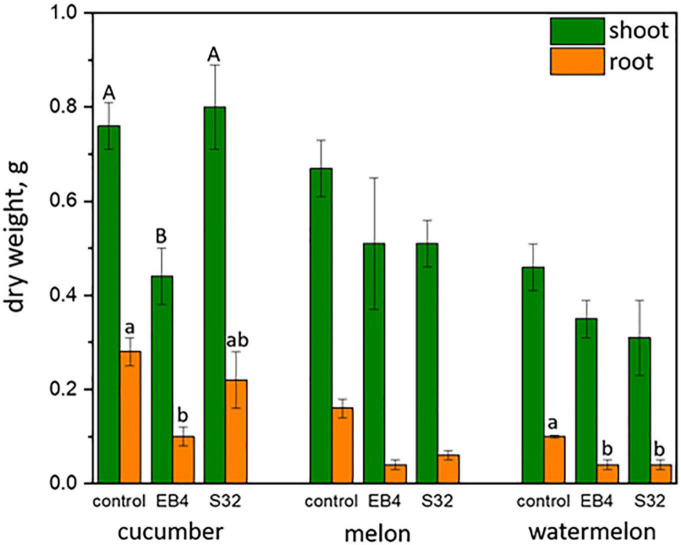
Shoot and root dry weight (mean ± standard error) of cucurbitaceous plants inoculated with *Diaporthe atlantica* strain EB4 and *Diaporthe iberica* strain S32. For each plant species and organ, letters on columns indicate significantly different means.

Neither dark lesions in roots or seedling wilt, symptoms reported for cucurbit root rot disease caused by *D. sclerotioides* (Shishido et al., 2006, 2014) were observed in plants inoculated with *D. atlantica* strain EB4 or *D. iberica* strain S32. However, a significant (*P* < 0.05) inhibition of shoot and root growth occurred in cucumber inoculated with strain EB4, and in roots of melon and watermelon inoculated with strains EB4 and S32 (Figure 6).

## 4. Discussion

The association of *Diaporthe* species with many important plant diseases has stimulated a considerable interest in this genus. On the other hand, *Diaporthe* species are ubiquitous in nature as endophytes, and have been reported as dominant components of the microbiome of various plant species. Therefore, more efforts are still required to increase our understanding of their ecology and biology. In this study, a multi-locus phylogenetic analysis of endophytic *Diaporthe* strains associated with roots of *Festuca pruinosa*, a grass that lives in a very unhospitable habitat, revealed two new species, *Diaporthe atlantica* and *Diaporthe iberica*, and possibly a third undescribed species represented by *Diaporthe* sp. 1. Both species described in this study belong to the *Diaporthe sojae* species complex. This complex is composed of endophytes, saprophytes and pathogens, and is the largest of the thirteen species complexes of *Diaporthe* recently described by Norphanphoun et al. (2022).

Over the past decade, DNA sequence data have become paramount for the accurate definition of species boundaries, and the resolution of important taxonomic questions. In the genus *Diaporthe*, multi-locus phylogenies based on the combined analysis of five genes were found to be better suited for an accurate species delimitation than those based on four or less loci (Santos et al., 2017). Therefore, the five most commonly used loci (ITS, TUB, TEF1, CAL, and HIS) for the separation of species in this genus were used in the present study to resolve the taxonomy of endophytic *Diaporthe* strains from *Festuca pruinosa* (Gomes et al., 2013; Gao et al., 2017; Guarnaccia et al., 2018; Guo et al., 2020; Norphanphoun et al., 2022). Among the *Diaporthe* species identified, *Diaporthe atlantica* was the most abundant, occurred in roots of plants from all four locations examined, and its populations were genotypically diverse. Phylogenetically, *D. atlantica* was close to, but clearly differentiated from *D. sclerotioides*. However, the comparison of morphological characters such as the culture appearance, or the shape and size of alpha conidia make impossible to separate both species (Shishido et al., 2006). Indeed, the difficulties in morphological comparisons have historically made the taxonomy of *Diaporthe* very challenging (Gomes et al., 2013). *Diaporthe* species often have similar or overlapping morphological characteristics (Lawrence et al., 2015), and due to their plasticity and inability to properly reflect the evolutionary history of species, these cultural characteristics have been deemed unfit for species delimitation (Rehner and Uecker, 1994; Gao et al., 2015). Therefore, the five gene multi-locus phylogenetic analysis was the key approach used in the present study to classify accurately *Diaporthe atlantica* as new species.

The plants of *Festuca pruinosa* often grow in soilless fissures in the rock, where a compact mass of roots is associated to a complex microbiome (Pereira et al., 2019). In this habitat poor in nutrients, *D. atlantica* could be helping its host to survive by playing a role in the recycling of nutrients from dead Festuca roots and other organic debris. In connection with this, *D. atlantica* cultures had extracellular enzymatic activities capable of degrading protein to ammonium, cellulases which could reduce cell wall material to simpler carbohydrates, and siderophores which help to trap iron. Plant roots can readily absorb ammonium and some simple carbohydrates, thus, the acceleration of organic matter decay by this fungus could increase the nutrient availability in a nutrient poor environment. Therefore, in its host grass *D. atlantica* could be a latent saprophyte, an endophyte that becomes a saprophyte when host roots senesce or die. In addition, *Diaporthe* cultures produced IAA, which can stimulate root growth. This fungus is known to behave as a mutualist in other grasses than its original host, promoting leaf and root growth in tritordeum and perennial ryegrass (*Lolium perenne*) even under salinity conditions (Toghueo et al., 2022).

The other novel species identified in this study, *Diaporthe iberica*, was associated to two plant species inhabiting very different habitats. The strain T6 was isolated from roots of *Celtica gigantea*, a perennial grass that grows in nutrient-poor sandy soils in semiarid zones (Vázquez and Devesa, 1996; Vázquez de Aldana et al., 2021), while strains S32 and TH77 occurred in roots of the perennial grass *Festuca pruinosa* from sea cliffs. Like *D. iberica*, many *Diaporthe* species are known to be multi-host. For instance, *Diaporthe eres*, the type species of the genus has been reported as a pathogen and an endophyte in plant species belonging to several families (Udayanga et al., 2014a). *Diaporthe iberica* did not produce spores on any of the different agar media we tested. *Diaporthe* species like *D. endophytica* (Gomes et al., 2013), *D. infertilis* (Guarnaccia and Crous, 2017), and *D. parvae* (Guo et al., 2020) are also known to be sterile, at least under experimental conditions. Like *Diaporthe atlantica*, *D. iberica* could have a beneficial association with its original host, improving adaptability to nutrient poor environments by contributing to organic matter recycling. Amylase and cellulase activity, ammonium production from protein substrate, and siderophores were observed in its cultures. In addition, *Diaporthe iberica* strain T6 was previously found to exhibit the greatest plant growth promotion capability among 66 fungal endophytes from *Celtica gigantea*, increasing both leaf and root biomass, and the leaf content of several mineral elements in tritordeum (Vázquez de Aldana et al., 2021). Besides, we found that *D. iberica* strain S32 could significantly improve the growth of *Festuca pruinosa* clonal line CD8 in greenhouse conditions (data not shown).

In addition to *D. atlantica* and *D. iberica*, *Festuca pruinosa* hosts another undescribed species of *Diaporthe*, *Diaporthe* sp. 1. The single strain representing this taxon also showed *in vitro* characteristics consistent with potential plant growth promotion and nutrient recycling functions, like production of IAA, ammonium, siderophores, cellulase and phosphate solubilization. However, following the recommendations of the International Commission on the Taxonomy of Fungi on the description of new fungal species, the taxonomic status of this strain will have to await further collection, since only one member of this putative species is known so far (Aime et al., 2021).

As above mentioned, virulent *Diaporthe* pathogens can also occur as endophytes in alternative host plant species (Crous and Groenewald, 2005; Gomes et al., 2013). Because of the close relatedness of *Diaporthe atlantica* to *D. sclerotioides*, a well-known pathogen of cucurbits (Shishido et al., 2006), the potential pathogenicity of *D. atlantica* strain EB4 and *D. iberica* strain S32 were investigated on cucumber, melon and watermelon. None of these caused root necrosis or wilt symptoms on cucurbits, as reported for black root rot disease caused by *D. sclerotioides* on these species (Shishido et al., 2006, 2014). However, strains EB4 and S32 caused a decrease in the shoot and root biomass of the three cucurbits. This potentially harmful effect observed in cucurbits contrasts with the beneficial effects observed in plants of tritordeum, perennial ryegrass and tomato inoculated with the same *D. atlantica* strain (Pereira, 2021; Toghueo et al., 2022). A *Diaporthe iberica* strain also showed a beneficial response on tritordeum (Vázquez de Aldana et al., 2021). Therefore, it seems that the benefits imparted by *D. atlantica* and *D. iberica* may be host-dependent, with only particular plant–fungus interactions resulting in positive plant responses (Brader et al., 2017). The host plant species and genotype has been reported to have a significant influence on the symbiotic lifestyles of some *Colletotrichum* species, favoring in some cases a mutualistic, commensal, or pathogenic lifestyle (Redman et al., 2001). Further investigations in this direction are required to understand the bases of the change in lifestyle by these *Diaporthe* species.

## 5. Conclusion

This study revealed that at least three *Diaporthe* species are associated to roots of *Festuca pruinosa* as endophytes. We identified two new *Diaporthe* species, *Diaporthe atlantica*, *Diaporthe iberica* and a yet undescribed taxon *Diaporthe* sp.1. *Diaporthe atlantica* seems to be the most abundant taxon in *Festuca pruinosa*, while *D. iberica* is a multihost endophyte naturally associated to roots of at least two different species of grasses (*Festuca pruinosa* and *Celtica gigantea*) growing in very different habitats, marine sea cliffs and sandy soils in semiarid inland zones. We propose that these symbiotic fungi might improve nutrient availability for their hosts, living in a habitat where nutrient availability is very limited. Since some *Diaporthe* species are multihost, other plants sympatric with *Festuca pruinosa* in sea cliff habitats, like *Armeria maritima* or *Crithmum maritimum* (Lopez-Bedoya and Pérez-Alberti, 2009), might also be hosts of these *Diaporthe* species. However, the ecological role of the newly described species as well as their interaction with host plants still needs to be better understood. Descriptions, molecular data, *in vitro* activities, and host interactions of the *Diaporthe* species described in the present study could represent an important resource for agricultural scientists, plant pathologists and taxonomists.

## Data availability statement

The datasets presented in this study can be found in online repositories. The names of the repository/repositories and accession number(s) can be found in the article/Supplementary material.

## Author contributions

RT performed experiments, analyzed the data, and wrote the manuscript. IZ and BV supervised the research and wrote the manuscript. All authors designed the experiments, analyzed the data, and read and approved the final manuscript.
