# Supplementary material for: Diaporthe species associated with the maritime grass Festuca rubra subsp. pruinosa
Source: Front Microbiol. 2023 Feb 16;14:1105299. doi: 10.3389/fmicb.2023.1105299 (PMC9978114; doi:10.3389/fmicb.2023.1105299)
Supplement: Supplementary file 1 [file Data_Sheet_1.docx]

Supplementary Material

**Supplementary Figure 1.** Maximum likelihood tree of the combined five-gene alignment of 22 *Diaporthe* isolates with 243 sequences data from 95 *Diaporthe* species based on Hasegawa-Kishino-Yano model. The percentage of trees in which the associated taxa clustered together is shown next to the branches. The scale bar represent the number of substitutions per site. Type strains are in bold and strains from this study in green.

**Supplementary Figure 2.** Molecular Phylogenetic analysis by Maximum Likelihood method of the ITS sequence based on the Kimura 2-parameter model. Type strains are in bold and strains from this study in green.

**Supplementary Figure 3.** Molecular Phylogenetic analysis by Maximum Likelihood method of the *TUB* sequences based on the Hasegawa-Kishino-Yano model. Type strains are in bold and strains from this study in green.

**Supplementary Figure 4.** Molecular Phylogenetic analysis by Maximum Likelihood method of *TEF1* sequences based on the Kimura 2-parameter model. Type strains are in bold and strains from this study in green.

**Supplementary Figure 5.** Molecular Phylogenetic analysis by Maximum Likelihood method of *CAL* sequences based on the Tamura 3-parameter model. Type strains are in bold and strains from this study in green.

**Supplementary Figure 6.** Molecular Phylogenetic analysis by Maximum Likelihood method of *HIS* sequences based on the Tamura-Nei model. Type strains are in bold and strains from this study in green.

**Supplementary Figure 7.** The evolutionary history if the ITS gene region of *Diaporthe atlantica* strains and *Diaporthe sclerotioides* isolates inferred by the Maximum Likelihood method based on the Kimura 2-parameter model. The tree with the highest log likelihood (-664.12) is shown. The percentage of trees in which the associated taxa clustered together is shown next to the branches. The scale bar represent the number of substitutions per site. Type strain are in bold and strains from this study in green.


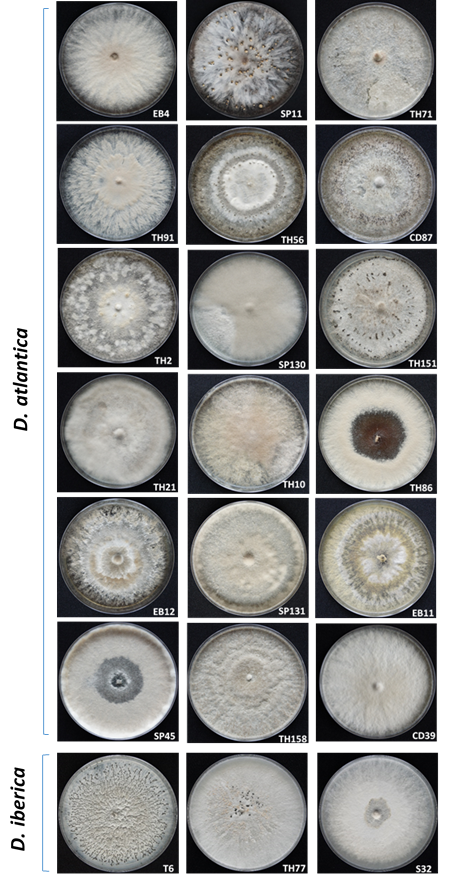


**Supplementary Figure 8.** Mosaic figure of *Diaporthe atlantica* and *Diaporthe iberica* strains

**Supplementary Tables**

**Supplementary Table 1**. Oligonucleotide primers and PCR amplification conditions used in the multilocus analysis of *Diaporthe* isolates.

| Target gene | Primer | Sequence (5'→3') | Amplification conditions | References |
| --- | --- | --- | --- | --- |
| **ITS** | **ITS 4** | 5'-TCCTCCGCTTATTGATATGC-3' | 94°C: 2min, (94°C: 30s, 58°C: 1min, 72°C: 1min) x 40 cycles, 72°C: 3min and cool down at 4°C | White et al. (1990) |
|  | **ITS 5** | 5'-GGAAGTAAAAGTCGTAACAAGG-3' |  |  |
| ***TUB*** | **T1** | 5'-AACATGCGTGAGATTGTAAGT-3' | 94°C: 2min, (94°C: 30s, 55°C: 1min, 72°C: 1min) x 40 cycles, 72°C: 3min and cool down at 4°C | O’Donnell and Cigelnik (1997) |
|  | **Bt-2b** | 5'-ACCCTCAGTGTAGTGACCCTTGGC-3' |  | Glass and Donaldson (1995) |
| ***CAL*** | **CAL-228F** | 5'-GAGTTCAAGGAGGCCTTCTCCC-3' | 94°C: 2min, (94°C: 30s, 58°C: 1min, 72°C: 1min) x 40 cycles, 72°C: 3min and cool down at 4°C | Carbone and Kohn (1999) |
|  | **CAL-737R** | 5'-CATCTTCTGGCCATCATGG-3' |  |  |
| ***TEF1*** | **EF1-728F** | 5'-CATCGAGAAGTTCGAGAAGG-3' | 94°C: 2min, (94°C: 30s, 55°C: 1min, 72°C: 1min) x 40 cycles, 72°C: 3min and cool down at 4°C | Carbone and Kohn (1999) |
|  | **EF1-986R** | 5'-TACTTGAAGGAACCCTTACC-3' |  |  |
| ***HIS*** | **CYLH3F** | 5'-AGGTCCACTGGTGGCAAG-3' | 94°C: 2min, (94°C: 30s, 58°C: 1min, 72°C: 1min) x 40 cycles, 72°C: 3min and cool down at 4°C | Crous et al. (2004) |
|  | **H3-1b** | 5'-GCGGGCGAGCTGGATGTCCTT-3' |  | Glass and Donaldson (1995) |

*CAL*: partial sequence of the gene coding for calmodulin; *HIS*: Histone H3; ITS: rDNA internal transcribed spacer region; *TEF1*: partial sequence of the gene coding for translation elongation factor 1-α; *TUB*: partial sequence of the gene coding for β-tubulin.

**Supplementary Table 2.** Single locus haplotypes detected by sequencing five nuclear loci of *Diaporthe atlantica* strains (*n* = 18). Dots denote sites with the same nucleotide variant to that of the first sequence, and dashes denote gaps.

**Table S2a.** Internal transcribed spacers and the 5.8 region of the nuclear ribosomal DNA (ITS).

| Haplotype | N | Position on alignment (bp) | |
| --- | --- | --- | --- |
|  |  | 335 | 364 |
| ITS-H1 | 16 | T | A |
| ITS-H2 | 2 | C | G |

**Table S2b.** Translation elongation factor 1-alpha (*tef1-α*).

| Haplotype | N | Position on alignment (bp) | | | | | | | | | | | | | | | | | |
| --- | --- | --- | --- | --- | --- | --- | --- | --- | --- | --- | --- | --- | --- | --- | --- | --- | --- | --- | --- |
|  |  | 18 | 20 | 21 | 23 | 26 | 27 | 31 | 32 | 34 | 36 | 41 | 43 | 47 | 61 |  |  |  |  |
| *tef1*-H1 | 1 | G | G | C | A | T | A | C | G | T | G | T | T | T | G |  |  |  |  |
| *tef1*-H2 | 1 | . | . | . | . | . | . | . | . | . | . | G | . | . | T |  |  |  |  |
| *tef1*-H3 | 3 | . | C | . | G | . | . | . | . | G | T | . | . | G | . |  |  |  |  |
| *tef1*-H4 | 4 | . | C | . | G | . | C | . | . | G | T | . | . | G | . |  |  |  |  |
| *tef1*-H5 | 1 | A | . | . | . | C | . | A | . | . | . | G | C | . | T |  |  |  |  |
| *tef1*-H6 | 1 | . | . | G | . | C | . | . | . | . | . | . | . | G | . |  |  |  |  |
| *tef1*-H7 | 1 | . | . | . | . | . | . | . | . | G | T | . | . | . | T |  |  |  |  |
| *tef1-*H8 | 3 | . | . | . | . | . | . | . | . | . | . | G | . | . | . |  |  |  |  |
| *tef1-*H9 | 1 | . | C | . | G | . | T | . | . | G | T | . | . | G | . |  |  |  |  |
| *tef1-*H10 | 1 | . | C | . | G | . | C | . | C | G | T | . | . | G | . |  |  |  |  |
| *tef1-*H11 | 1 | . | . | . | . | . | . | . | . | . | T | . | . | . | . |  |  |  |  |

**Table S2c.** Histone H3 (*his*).

| Haplotype | N | Position on alignment (bp) | | | | | | | | | | | | | | | | | |
| --- | --- | --- | --- | --- | --- | --- | --- | --- | --- | --- | --- | --- | --- | --- | --- | --- | --- | --- | --- |
|  |  | 77 | 212 | 309 | 312 | 315 | 321 | 325 | 355 | 415 | 416 |  |  |  |  |  |  |  |  |
| *his*-H1 | 3 | C | C | C | G | T | C | G | C | C | C |  |  |  |  |  |  |  |  |
| *his-*H2 | 1 | . | A | . | . | C | . | T | T | A | . |  |  |  |  |  |  |  |  |
| *his*-H3 | 1 | . | A | . | . | C | . | . | . | . | . |  |  |  |  |  |  |  |  |
| *his*-H4 | 2 | . | A | T | . | . | T | . | . | . | . |  |  |  |  |  |  |  |  |
| *his*-H5 | 2 | . | . | . | . | . | . | . | . | . | T |  |  |  |  |  |  |  |  |
| *his*-H6 | 5 | . | A | T | . | . | . | . | . | . | . |  |  |  |  |  |  |  |  |
| *his*-H7 | 2 | . | . | . | T | . | . | . | . | . | . |  |  |  |  |  |  |  |  |
| *his-*H8 | 1 | . | A | . | . | . | . | . | . | . | . |  |  |  |  |  |  |  |  |
| *his-*H9 | 1 | T | A | T | . | . | . | . | . | . | . |  |  |  |  |  |  |  |  |

**Table S2d.** β-tubulin (*TUB*).

| Haplotype | N | Position on alignment (bp) | | | | | | | | | | | | | | | | | |
| --- | --- | --- | --- | --- | --- | --- | --- | --- | --- | --- | --- | --- | --- | --- | --- | --- | --- | --- | --- |
|  |  | 63 | 198 | 253 | 315 | 578 | 598 | 693 |  |  |  |  |  |  |  |  |  |  |  |
| *tub*-H1 | 4 | G | G | T | C | C | T | T |  |  |  |  |  |  |  |  |  |  |  |
| *tub-*H2 | 1 | T | . | . | . | . | . | . |  |  |  |  |  |  |  |  |  |  |  |
| *tub*-H3 | 2 | . | A | . | T | . | . | . |  |  |  |  |  |  |  |  |  |  |  |
| *tub*-H4 | 2 | . | A | . | . | T | . | C |  |  |  |  |  |  |  |  |  |  |  |
| *tub*-H5 | 5 | . | A | . | . | . | . | . |  |  |  |  |  |  |  |  |  |  |  |
| *tub*-H6 | 2 | . | . | C | . | . | C | . |  |  |  |  |  |  |  |  |  |  |  |
| *tub*-H7 | 2 | . | A | . | . | T | . | . |  |  |  |  |  |  |  |  |  |  |  |

**Table S2e.** Calmodulin (*CAL*).

| Haplotype | N | Position on alignment (bp) | |
| --- | --- | --- | --- |
|  |  | 161 | 176 |
| *cal*-H1 | 5 | T | A |
| *cal*-H2 | 11 | G | C |
| *cal*-H3 | 2 | G | . |

**Supplementary Table 3.** Summary of biochemical characteristics of *Diaporthe* strains.

| ***Diaporthe* species** | **Strain** | **IAA (µg/mL)** | **Siderophores (cm)** | **NH4**  **(µM)** | **Phosphate solubilization (cm)** | **Fungal growth diameter (cm)** | | | **Enzymes** | | |
| --- | --- | --- | --- | --- | --- | --- | --- | --- | --- | --- | --- |
|  |  |  |  |  |  | **0 mM** | **300 mM** | **600 mM** | **A** | **P** | **C** |
| ***D. iberica*** | T6 | 1.074 | 1.500 | 161.964 | 0.000 | 5.825 | 3.500 | 1.975 | **+** | **-** | **+** |
|  | S32 | 6.287 | 3.050 | 367.685 | 0.000 | 7.275 | 5.000 | 3.600 | **+** | **-** | **+** |
|  | TH77 | 3.291 | 1.700 | 309.561 | 1.150 | 5.225 | 3.400 | 2.175 | **-** | **+** | **+** |
|  |  |  |  |  |  |  |  |  |  |  |  |
| ***D. atlantica*** | EB11 | 6.788 | 2.100 | 47.675 | 3.850 | 3.125 | 1.750 | 1.025 | **+** | **+** | **+** |
|  | TH21 | 1.867 | 2.450 | 332.092 | 0.000 | 5.475 | 5.100 | 2.825 | **-** | **-** | **+** |
|  | TH71 | 0.288 | 2.400 | 481.321 | 0.000 | 5.025 | 4.100 | 2.400 | **-** | **-** | **+** |
|  | SP130 | 9.186 | 2.100 | 276.580 | 3.250 | 4.600 | 4.300 | 2.950 | **+** | **-** | **+** |
|  | TH158 | 26.708 | 2.950 | 133.555 | 2.050 | 4.150 | 5.500 | 3.675 | **+** | **-** | **+** |
|  | CD87 | 9.121 | 1.800 | 244.252 | 1.525 | 3.075 | 2.875 | 4.125 | **-** | **-** | **+** |
|  | TH151 | 8.489 | 2.050 | 66.614 | 1.250 | 3.150 | 3.500 | 2.600 | **-** | **-** | **+** |
|  | TH56 | 12.321 | 0.000 | 568.834 | 0.000 | 5.625 | 3.550 | 2.825 | **+** | **-** | **+** |
|  | TH91 | 42.316 | 2.800 | 115.269 | 2.875 | 6.600 | 5.225 | 3.600 | **+** | **-** | **+** |
|  | CD39 | 12.102 | 2.300 | 195.598 | 1.975 | 2.625 | 1.900 | 2.100 | **+** | **-** | **+** |
|  | TH10 | 18.106 | 2.650 | 121.146 | 2.650 | 3.550 | 1.725 | 1.900 | **+** | **-** | **+** |
|  | SP45 | 71.584 | 2.200 | 240.660 | 0.000 | 6.275 | 7.025 | 3.475 | **-** | **-** | **-** |
|  | TH86 | 17.056 | 2.050 | 454.545 | 3.825 | 7.100 | 4.950 | 3.325 | **-** | **+** | **+** |
|  | EB4 | 60.280 | 2.850 | 110.697 | 0.000 | 3.100 | 2.075 | 1.125 | **+** | **+** | **+** |
|  | TH2 | 17.480 | 0.000 | 158.046 | 2.000 | 4.450 | 1.525 | 2.000 | **+** | **-** | **+** |
|  | SP11 | 13.851 | 2.400 | 124.412 | 1.300 | 5.925 | 3.800 | 2.675 | **-** | **-** | **+** |
|  | EB12 | 15.630 | 2.400 | 82.941 | 1.750 | 3.025 | 3.900 | 1.400 | **+** | **-** | **-** |
|  | SP131 | 7.938 | 1.700 | 137.473 | 2.475 | 4.125 | 1.550 | 2.450 | **+** | **-** | **+** |
|  |  |  |  |  |  |  |  |  |  |  |  |
| ***Diaporthe* sp.1** | EB73 | 1.418 | 2.250 | 136.167 | 1.350 | 6.075 | 4.725 | 1.950 | **-** | **+** | **+** |

Extracellular enzymes: amylase (A), cellulase (C), protease (P). Fungal growth was tested at different NaCl concentrations: 0mM, 300mM and 600mM.
