# Supplementary material for: Diaporthe species associated with the maritime grass Festuca rubra subsp. pruinosa
Source: Front Microbiol. 2023 Feb 16;14:1105299. doi: 10.3389/fmicb.2023.1105299 (PMC9978114; doi:10.3389/fmicb.2023.1105299)

## Slide 1
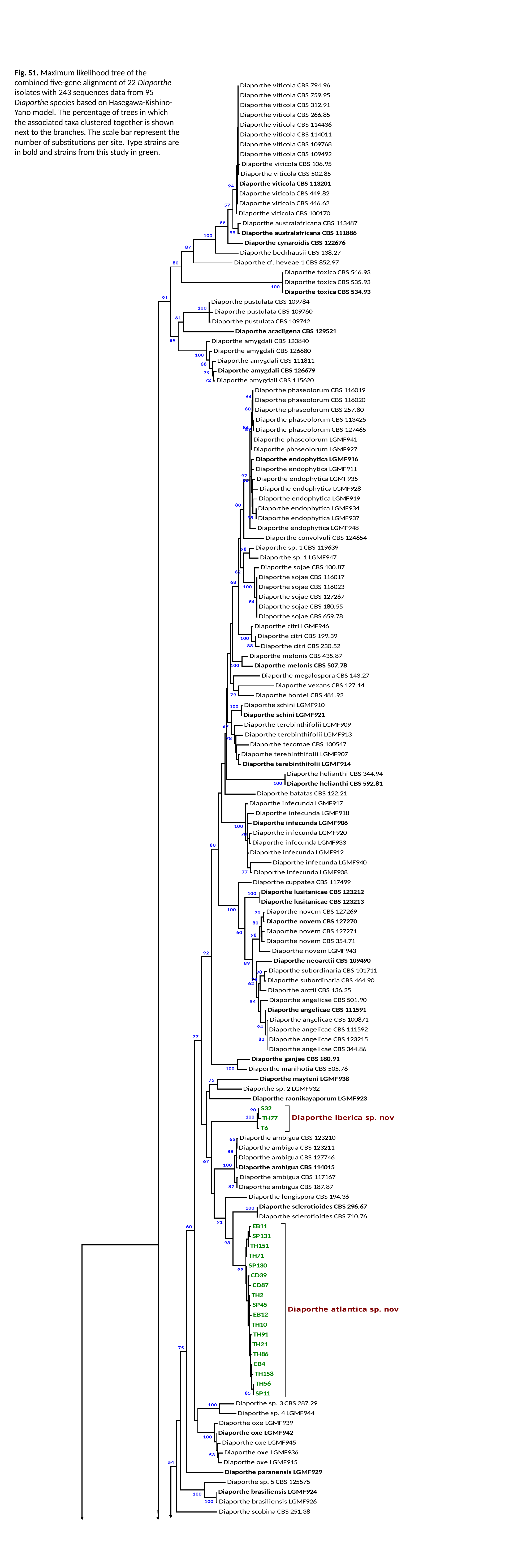

Fig. S1. Maximum likelihood tree of the combined five-gene alignment of 22 Diaporthe isolates with 243 sequences data from 95 Diaporthe species based on Hasegawa-Kishino-Yano model. The percentage of trees in which the associated taxa clustered together is shown next to the branches. The scale bar represent the number of substitutions per site. Type strains are in bold and strains from this study in green.

## Slide 2
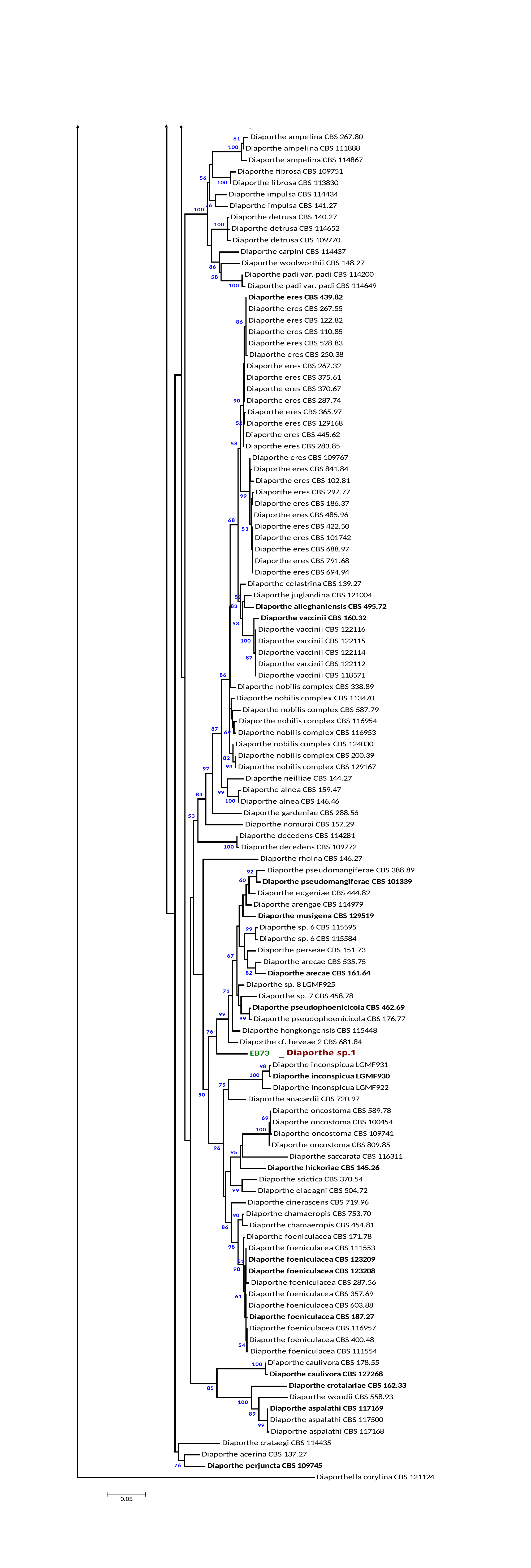

Supplement: Supplementary file 2 [file Presentation_1.PPTX]
